# New Carbonate-Based Materials and Study of Cytotoxic Capacity in Cancer Cells

**DOI:** 10.3390/ijms24065546

**Published:** 2023-03-14

**Authors:** Nayara Niza-Pérez, Josefa Quiroz-Troncoso, Nicolás Alegría-Aravena, Santiago Gómez-Ruiz, Diana Díaz-García, Carmen Ramírez-Castillejo

**Affiliations:** 1 COMET-NANO Group, Departamento de Biología y Geología, Física y Química Inorgánica, ESCET, Universidad Rey Juan Carlos, 28933 Madrid, Spain; 2 Centro de Tecnología Biomédica (CTB), Departamento Biotecnología-B.V. ETSIAAB, Universidad Politécnica de Madrid, 28223 Madrid, Spain; 3 Medicinal Gardens S.L. (Baïa Food), 28008 Madrid, Spain; 4 Grupo de Biología y Producción de Cérvidos, Instituto de Desarrollo Regional, Universidad de Castilla-La Mancha, 02006 Albacete, Spain; 5 Asociación Española Contra el Cáncer (AECC)—Fundación Científica AECC, 02001 Albacete, Spain; 6 Oncology Department, Instituto de Investigación Sanitaria San Carlos (IdISSC), 28040 Madrid, Spain

**Keywords:** glioblastoma, breast cancer, vaterite, manganese, cysteine

## Abstract

Calcium carbonate, one of the most commonly found biominerals produced by organisms, has shown great potential for the development of systems with biological applications due to its excellent biocompatibility, biodegradability, and simple chemical composition. Here, we focus on the synthesis of various carbonate-based materials with vaterite phase control and their subsequent functionalization for applications in treating glioblastoma, one of the most limiting tumors currently without effective treatments. The incorporation of l-cysteine into the systems increased cell selectivity while the incorporation of manganese supplied the materials with cytotoxic capacity. Extensive characterization of the systems by infrared spectroscopy, ultraviolet-visible spectroscopy, X-ray diffraction, X-ray fluorescence, and transmission electron microscopy confirmed the incorporation of the different fragments causing selectivity and cytotoxicity to the systems. To verify their therapeutic activity, the vaterite-based materials were tested in the CT2A cell line (murine glioma) and compared to SKBR3 (breast cancer) and HEK-293T (human kidney) cell lines. These studies on the cytotoxicity of the materials have shown promising results that can encourage future in vivo studies in glioblastoma models.

## 1. Introduction

After cardiovascular diseases, cancer is the second-leading cause of mortality worldwide [1]. Glioblastomas multiforme (GBMs) are the most frequent and aggressive brain tumors, with average life expectancy between 1 and 3 years from the time of diagnosis. They make up around 14% of all primary brain tumors and are more frequently developed in men than in women (1.6:1) [2,3]. The current treatment of GBMs is based on surgery, to safely remove as much of the tumor as possible, and radiotherapy and/or chemotherapy, in those cases where tumors cannot be surgically removed, to delay tumor growth. However, radio- and chemotherapy are not very effective because the resistance of the tumor cells and the high toxicity of the treatment, especially the latter, considerably reduce the patient’s quality of life. As a result, the development and introduction of novel drugs that are more effective and present fewer side effects have become an urgent problem regarding the treatment of GBM [2,3,4].

Vaterite is a crystalline phase of calcium carbonate (CaCO_3_) and a polymorph of calcite and aragonite. It is used as a biocompatible carrier in an increasing number of applications in biomedicine thanks to its particle porosity, safety profile, low cost, high mechanical stability, and high solubility, which allows controlled drug release [5,6]. Vaterite dissolves rapidly at acidic pH, so it can undergo degradation both in vitro and in vivo [7,8,9].

Cysteine is an important component of most cellular proteins and lipids. Rapidly growing cancer cells need large amounts of this amino acid to produce new cells and to protect themselves from the natural toxic peroxides that are produced due to their highly active metabolism [10]. Several studies have already used cysteine or its derivatives as target molecules in order to increase the cytotoxicity of the drugs used by increasing selectivity and accumulation in the target area [11,12]. The relevance of cysteine metabolism in cancer has gained interest in recent years due to the different mechanism of action, in which cysteine plays an important role. For example, it is involved in glutathione generation, playing an antioxidant role, but it is also involved in the production of hydrogen sulfide, a necessary food in the mitochondrial electron transfer chain [13]. More specifically, some studies have found that cysteine catabolism results in the accumulation of cysteine sulfonic acid in GBM, highlighting its ability to inhibit oxidative phosphorylation (decreased cellular respiration) in this cell type [14]. In short, cancer cells depend on cysteine accumulation to promote proliferation in selective form, and produce redox interrupters for enzyme modifications and better antioxidant capacity [15].

Manganese, a chemical element belonging to the transition metals block of the periodic table, is a trace element necessary for growth and development. Manganese oxide nanoparticles (MnO NPs) have been discovered to exhibit strong cytotoxicity in in vitro studies against human glioblastoma cell lines [16]. Additionally, these manganese-based cell treatments result in an increase in reactive oxygen species (ROS), which, in turn, stimulates the activity of the caspase-3 enzyme and induces chromatin condensation and breakdown, signaling the beginning of the apoptotic process in cells [17,18].

To date, there are no studies based on the functionalization of vaterites with application in glioblastoma; therefore, this work focuses on the synthesis of various materials based on carbonates and their subsequent functionalization with different amounts of L-cysteine. The aim of the present investigation is to increase cell selectivity and manganese incorporation into the carbonate-based systems in order to prepare materials with high cytotoxic capacity against cancer cells with special consideration of GBM cell lines, due to the limited number of treatments that are active against this type of tumor. In addition, this study will be extended to other potential cell types, such as breast cancer, to determine whether the studied systems are able to be applied in different types of cancers.

## 2. Results

### 2.1. Materials Synthesis and Characterization

#### 2.1.1. Synthesis of Vaterite Materials and Its Functionalizations

**Scheme 1:**
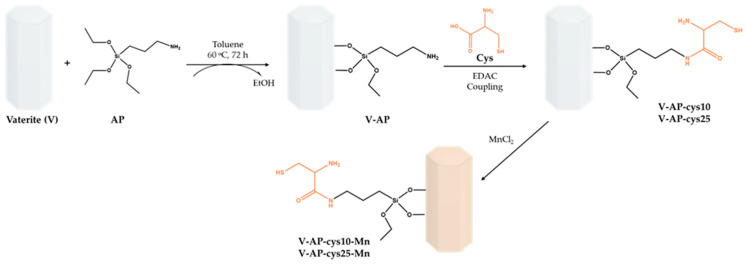
General scheme of the synthesis of vaterite materials.

Synthesis of the starting vaterite was carried out through a precipitation reaction starting from CaCl_2_ and (NH_4_)_2_CO_3_. Subsequently, the covalent incorporation of the 3-aminopropyltriethoxysilane (AP) ligand was carried out through a protonolysis reaction (Scheme 1). The incorporation of the amino acid cysteine at two different concentrations (10% and 25% by mass) was carried out by amidation through EDAC coupling (Scheme 1).

For the incorporation of manganese into vaterite, two types of synthesis were carried out (see Section 4). The one-pot method was based on the incorporation of Mn^2+^ ions during the formation of vaterite, and the post-synthesis method was based on incorporating manganese into a series of cysteine-containing materials (V-AP-cys10 and V-AP-cys25).

#### 2.1.2. Infrared Spectroscopy (IR)

Synthesized materials were characterized by infrared spectroscopy. The infrared spectra of the different series showed bands typically associated with calcite and vaterite, confirming the materials are composed of a mixture of these crystalline phases of CaCO_3_. 

**Figure 1:**
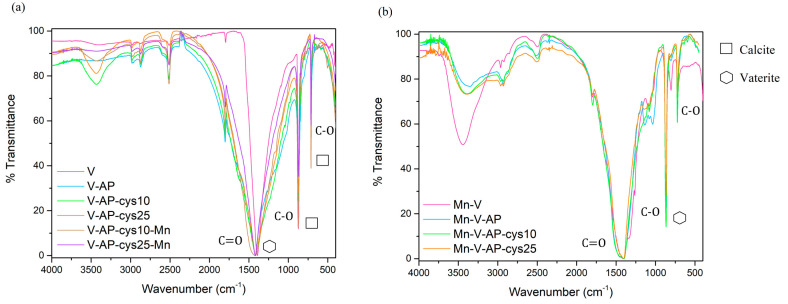
Comparative FT-IR spectra: (**a**) V Series, (**b**) Mn-V Series.

These characteristic bands are marked in Figure 1. At wavelengths 713 cm^−1^ and 873 cm^−1^ characteristic bands of calcite are observed, while at wavelengths 867 cm^−1^ and 1420 cm^−1^, characteristic bands of vibrational frequencies of CO_3_^2−^ of vaterite can be observed.

Manganese functionalized materials using the one-pot method (Mn-V materials) have bands at 720 cm^−1^ and 1400 cm^−1^ that do not coincide with the characteristic bands of any crystalline phase; this shift is probably due to the introduction of manganese.

These characteristic bands for each crystalline phase are maintained for each of the syntheses carried out, as well as after the different functionalizations carried out on the starting vaterite. The bands around 1400 cm^−1^ correspond to the C=O double bonds, while the bands around 715 and 870 correspond to the C-O bonds.

#### 2.1.3. Ultraviolet-Visible Spectroscopy (UV-Vis)

**Figure 2:**
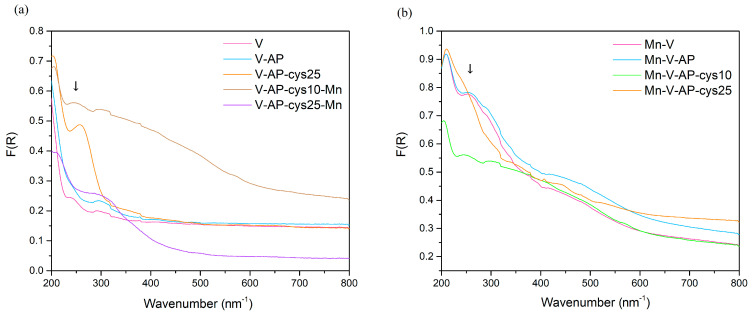
Comparative ultraviolet-visible spectra for (**a**) V series, (**b**) Mn-V series.

Synthesized materials were characterized by solid-state ultraviolet-visible spectroscopy, in order to detect the maximum absorption peaks of each material. The peak (arrow) shown in Figure 2 is characteristic of cysteine, indicating the correct incorporation of the amino acid into the carbonate material.

In the V series, the appearance of a peak around 260 nm can be clearly observed, while in the Mn-V series, it appears with less intensity, even remaining a single broad signal in the case of Mn-V-cys25.

#### 2.1.4. X-ray Diffraction (XRD)

**Figure 3:**
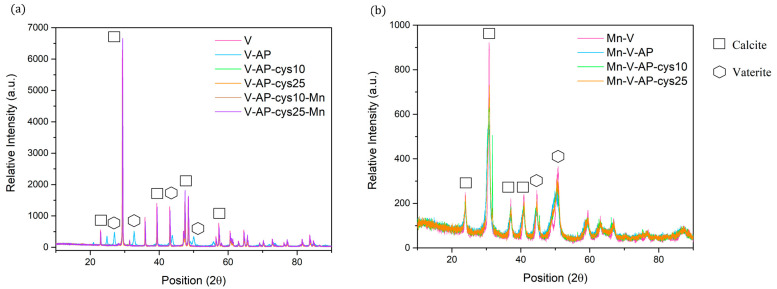
Comparative X-ray diffraction diffractogram for (**a**) V series, (**b**) Mn-V series.

Synthesized materials were characterized by X-ray diffraction. As in the case of IR spectroscopy, diffraction peaks typically belonging to calcite and vaterite were detected and are shown in Figure 3. We can appreciate an increase in the width of the peaks of the Mn-V series, which may be due to the introduction of Mn ions in the carbonate structure itself. The peak with the highest intensity belongs to calcite, so we can deduce that this phase is more present in the materials (78% of calcite, 28% of vaterite), which may be due to the low metastability of vaterite and the reaction conditions, since vaterite initiates its transition to calcite at temperatures below 60 °C, analogous to the starting vaterite (V) without manganese (83% of calcite, 17% of vaterite).

The crystallite size (D) of each material has been calculated through Scherrer’s formula, taking the most intense peak of each diffractogram (Table 1).

As can be seen in the table, the structure of the material is affected by the synthetic route followed. The post-synthesis route, in which the starting vaterite (V) is prepared and functionalized with cysteine and manganese in different synthetic steps, leads to the formation of crystallites of a much larger size (55 nm on average) than the materials starting from the vaterite with manganese in the one-pot (Mn-V) method (11 nm on average).

#### 2.1.5. X-ray Fluorescence (XRF)

Synthesized materials were characterized by XRF in order to determine the quantity of Mn and S, thus allowing for the calculation of cysteine in the system. The results show that materials with Mn introduced at the beginning (in a one-pot method during vaterite formation) of the series contain a greater amount of the metal. In contrast, the incorporation of Mn using the post-synthetic method of calcium carbonate results in lower functionalization. 

The amount of incorporated cysteine was higher, as expected, in materials prepared using 25% cysteine, according to the real percentages calculated by XRF from the concentration of sulfur present (Table 2). In all cases, it is important to note that the amount of cysteine was lower than the theoretical amount added in the EDAC coupling reaction.

#### 2.1.6. Transmission Electron Microscopy (TEM) and Dynamic Light Scattering (DLS)

**Figure 4:**
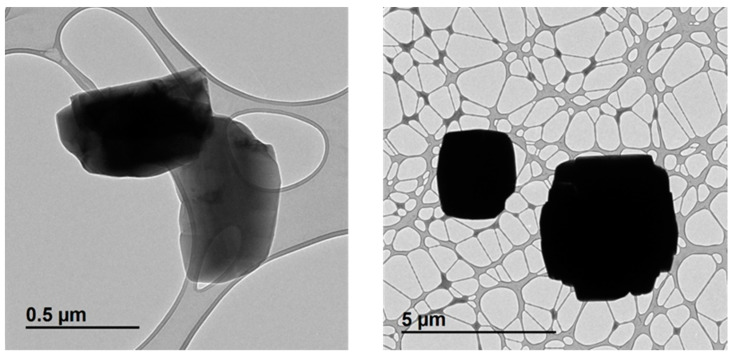
TEM micrographs of Vaterite at different magnifications.

A series of images of the structure of the vaterite material was obtained using the transmission electron microscope. As seen in Figure 4, these calcium carbonates measure about a micron in size and present a rectangular and homogeneous morphology forming quadrangular aggregates.

In order to determine the size of such designed systems, DLS studies were carried out using PBS buffer with pH 7.4. In Table 3, the calculated hydrodynamic sizes can be observed. The determined sizes are very similar to those observed in TEM micrographs, indicating good stability of the system in an aqueous medium with low aggregation. The starting materials (V and Mn-V) have a size around 1 micron and 1.5 microns, respectively, which indicates that the systems are very similar; nevertheless, the incorporation of Mn during the synthesis of vaterite seems to promote a slight vaterite structure growth. After several modifications of the starting vaterites by covalent functionalization (incorporation of the AP ligand and cysteine), no significant increase in the size of the structures was observed, as the average hydrodynamic size for V-AP-cys25 is very similar to the size of V and the hydrodynamic size of Mn-V-AP-cys25 is similar to that of Mn-V. Table 3 presents the dispersity index of the materials in the simulated biological medium (PBS pH 7.4), which shows a narrow distribution for all the measurements (with an average dispersity of around 1.3). These low dispersity values indicate that the materials do not undergo significant aggregation when dispersed in the studied aqueous medium, facilitating their use in biological applications.

### 2.2. Cell Toxicity Assay

**Figure 5:**
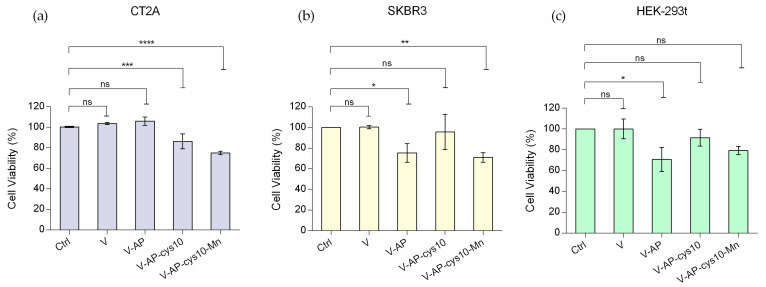
Variation in cell viability during the functionalization process of the starting material. Concentration of each tested product is 0.25 mg/mL with respect to vaterite. (**a**) Cell viability in CT2A cell line; (**b**) cell viability in SKBR3 cell line; (**c**) cell viability in HEK-293t cell line. The significant differences are indicated as **** *p* < 0.0001, *** *p* < 0.001, ** *p* < 0.01, * *p* < 0.05. (ns is not significant).

Cell viability assays were performed on glioblastoma cells (CT2A) to study the sensitivity that these cells presented to the synthesized compounds, contrasted to another cancer type (breast cancer, SKBR3) and non-cancerous cells (HEK-293T. Figure 5 shows the effect of these materials on the CT2A (Figure 5a), SKBR3 (Figure 5b), and HEK-293T (Figure 5c) cell lines. A small decrease in cell viability is observed after addition of cysteine; this is more noticeable after functionalization with manganese, with the last material having a higher activity (enhancing cell death).

**Figure 6:**
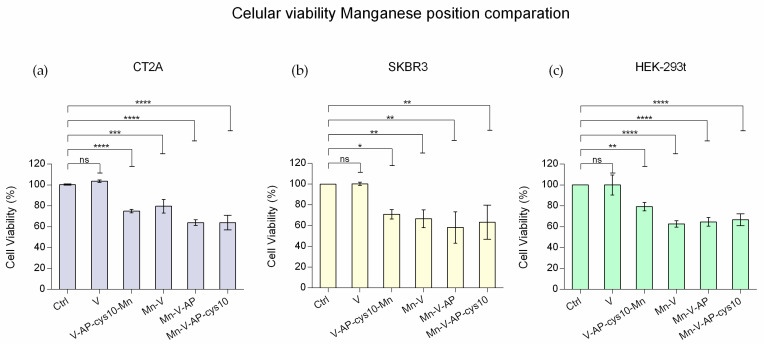
Variation in cell viability depends on the area of functionalization of the material with manganese. Concentration of each tested product is 0.25 mg/mL with respect to vaterite. (**a**) Cell viability in CT2A cell line; (**b**) cell viability in SKBR3 cell line; (**c**) cell viability in HEK-293t cell line. The significant differences are indicated as **** *p* < 0.0001, *** *p* < 0.001, ** *p* < 0.01, * *p* < 0.05, ns is not significant.

In order to verify the effectiveness of the materials depending on the placement of the element within the complex, we designed tests with the different incorporation positions of the elements of interest: vaterite, manganese, and cysteine. Figure 6 shows the change in cell viability using the materials prepared with the one-pot method in comparison with those prepared by using a post-synthesis method. Figure 6a shows the noticeable effect on GBM cells in a dose-dependent manner, with viability reaching around 50% in the case of the last material. The same behavior was found in breast cancer cells (Figure 6b), with the last material reaching around 40% viability. Figure 6c, shows the effects of these materials on non-tumoral cells, also reaching around 40% cell viability.

**Figure 7:**
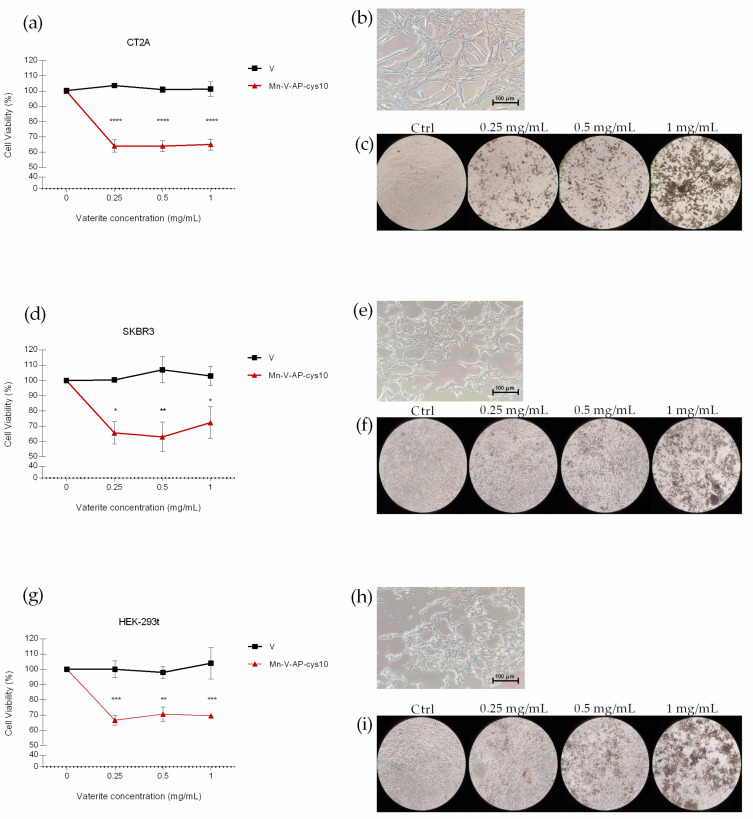
Cell viability at different concentrations (0, 0.25, 0.5, and 1 mg/mL) and saturation effect produced by precipitation of the materials. (**a**) Dose response in CT2A cells; (**b**) CT2A line cells in normal growth; (**c**) CT2A cells micrographies with different concentrations of vaterite. (**d**) Dose response in SKBR3 cells; (**e**) SKBR3 line cells in normal growth; (**f**) SKBR3 cells micrographies with different concentrations of vaterite. (**g**) Dose response in 293t cells; (**h**) 293t line cells in normal growth; (**i**) 293t cells micrographies with different concentrations of vaterite. The significant differences are indicated as **** *p* < 0.0001, *** *p* < 0.001, ** *p* < 0.01, * *p* < 0.05.

The synthetic systems were initially tested at three concentrations (0.25, 0.5, and 1 mg/mL of each material) compared to a culture medium control. An analysis of the different assays was performed, observing a tendency to the tangent of the cell survival curve. Starting from the 0.25 mg/mL concentration there does not appear to be a dose-dependent effect. This type of experimental curve indicates saturation phenomena, where the material no longer enters the cells due to there being an excess in the medium. This effect can be seen in Figure 7, looking at the different cell responses at the basal (vaterite) and better conditions (vaterite with manganese, AP, and cysteine to 10 mg/mL) (Figure 7a,d,g). Cells were treated at the optimum moment of growth, akin to other types of assays, when cell growth reached 70 to 80% confluence in the culture dish, as can be seen in the cell micrographs in Figure 7b (CT2A cells), Figure 7e (SKBR3 cells), and Figure 7h (HEK-293t cells). It is important to note that the same saturation effect that seems to take place with the increase in concentration can be observed in the precipitates that form in the cell culture plates, as seen in Figure 7c (CT2A cells), Figure 7f (SKBR3 cells), and Figure 7i (HEK-293t cells).

To demonstrate the capacity of selectivity (internalization) of the studied materials with cysteine as a directing agent, Mn-V-AP and Mn-V-AP-cys25 materials were incubated with the three cell lines for 72 h. After this time, the cells were crushed, and the intracellular content was measured by ICP to determine the amount of cell-internalized Mn (Table S1). The material without cysteine has an acceptable internalization capacity of between 22–33% in the studied cell lines. However, when cysteine is incorporated into the materials as a targeting molecule, internalization is increased up to twice that of the material without cys, with internalization percentages up to 53%.

## 3. Discussion

The use of biomaterials to fight diseases has become an important area in recent times, particularly for targeting GBM, with the characteristics of calcium carbonates, materials with an adequate biodegradable composition, particle size, and other structural features making them excellent drug carriers. 

To date, vaterite is described as one of the most stable phases of carbonate-based materials with excellent potential for use in therapeutic approaches [19]. Apart from a lack of toxicity, authors have shown that when loaded with various compounds, vaterite-based systems are able to maintain their activity after encapsulation [20]. 

In the present work, two sets of vaterite-based materials (using one-pot synthesis or post-synthesis methods) have been designed. These materials are functionalized with cysteine, providing selectivity to the material, and manganese, a cytotoxic agent. It is of note that the differences in the synthesis method determine the type of properties associated with each of the materials, with each prepared material undergoing thorough analysis using a variety of characterization techniques. The existence of a mixture of vaterite and calcite phases was confirmed via infrared spectroscopy and X-ray diffraction, showing calcite to be the predominant phase with percentages above 75%. (Figure 1 and Figure 3) [5], In addition, significant differences were observed in the average crystallite size of the materials of each series, with those prepared using the post-synthetic method being up to 5 times larger than those from the Mn-V series. This may be due to the fact that the materials in the post-synthesis series have vaterite (V) as the initial material, which is synthesized through a precipitation reaction without any other agent in the medium (such as MnCl_2_) that could hinder the growth of the vaterite crystals during their formation. XRF and UV-VIS spectroscopy techniques also confirmed the correct functionalization of the materials with the different fragments (Figure 2) [19,21]

These observed differences led us to check the innocuity of the vaterite (V), as is shown in Figure 5c, Figure 6c, and Figure 7g. Here, vaterite at concentrations ranging from 0.25 to 1 mg/mL did not have any effect on the tested cell lines, without a material-dependent effect. In this sense, its use can be optimized through studies at doses lower than this value. However, high concentrations of the compound produced a flattening effect and complicated cell growth, as is shown in Figure 7c,f,i. Multiple authors describe the low solubility and potential saturation of the medium of these compounds, particularly in microbial cultures [22,23]. These high concentrations produce osmotic problems, high viscosity in the medium, and low oxygen transport. Therefore, our work focused on low concentrations of vaterite.

When functionalizing vaterite with the 3-aminopropyltriethoxysilane (V-AP) ligand, the compound shows no apparent anticancer activity in the CT2A cell line (Figure 5a) but is able to reduce cellular viability by close to 20% in SKBR3 cells (Figure 5b). These different effects could be due to the fact that the AP ligand generates cell type selectivity, which has been previously discussed for other therapeutic systems studied in cytotoxic experiments [24,25]. However, an increased effect is seen when the molecule is coupled with cysteine, possibly due to the cancer cell’s affinity for this amino acid and the proliferation and survival effect produced by this molecule. In those cases where cysteine was coupled with different molecules that have contact with cancer cells, there was a decrease in cell viability [15].

When analyzing cell toxicity, materials prepared using the post-synthetic method have less therapeutic activity than materials prepared using the one-pot synthetic method, shown in Figure 6. This could be because the greater amount of Mn in this material provides easier access to the Mn^2+^ ions in the carbonate structure with the one-pot synthetic method. Another possibility is that treatment induces apoptosis in tumoral cells, coinciding with a previous study in prostate cancer cells where Mn reduces cell viability and promotes the G0/G1 phase in these cells [26]. 

On the other hand, in the post-synthetic materials, the surface of the material seems to be saturated by the previous incorporation of AP and cys, thus not allowing Mn loading to the same degree. We have verified that the optimal concentration for high activity of in vitro cultures is 0.25 mg/mL of vaterite together with Mn, as shown in Figure 7a,d,g. In addition, the cysteine effect has also been verified, since materials functionalized with the amino acid caused greater cell death than those without the organic fragment. This improvement in the cytotoxic effect even rises above 50% when a combination of manganese with cysteine is used, indicating the importance of Mn capture during synthesis. The intracellular analysis in terms of manganese in all the cell lines studied showed that the incorporation of cysteine as a direct agent improved the internalization capacity of the materials up to 2.3 times more than the materials that did not contain this amino acid. The results demonstrate that even low percentages of the amino acid allow the materials to acquire the ability to act selectively [27].

A similar effect can be seen in breast cancer. SKBR3 cells overexpress the HER2/c-erb-2 gene product, with this being the breast cancer tumor subtype with the highest growth rate and the most resistance to treatments until the appearance of the HER2 inhibitor. Using the treatment proposed here, we achieved high rates of sensitization of this resistant line. It is noteworthy that the same rates of sensitization are achieved in the recalcitrant CT2A glioblastoma cells.

In addition, the materials tested on healthy cells (HEK-293T cell line) unfortunately produce similar effects to chemotherapy, although this does not invalidate their usefulness (Figure 5c). Although an effect is seen in tumor cells (Figure 6a,b), there is also toxicity in non-tumor cells (Figure 6c), which can imply toxicity in the metabolization and elimination systems of the material, which is comparable to current medicine. However, the first objective of this work was to observe the better capacity for selectivity of cell death in tumor cells. A new front may be the use of nanoparticles to establish the best cell selection [28].

In addition, tumoral and normal cells fundamentally differ in their resilience to oxidative stress situations, which could explain the differential effect of the treatment on both populations. However, after further analysis, this does not appear to be the case. Instead, this effect could be attributed to high effectivity of the studied materials against an active cell cycle, as non-tumor cells typically present a slowed-down cycle, thus providing a possible explanation for the slight differences observed. HEK293T cells, although not considered tumoral cells, are immortalized cells with constant division in in vitro culture, unlike non-tumoral cells in vivo; thus, 293T cells may not be the ideal model. To counteract this possibility, we intend to test these materials in primary cell cultures in the future, despite their more difficult expansion in vitro. This would hypothetically allow for better discussion of the differences in cell viability of the treatments with vaterite and manganese as regards their cytotoxicity in tumor cells. Therefore, future studies will focus on testing the reported systems against primary cultures of different cell types before moving one step further to in vivo tests in animal models and establishing the best cell selection by nanoparticles.

## 4. Materials and Methods

### 4.1. Carbonate Synthesis

A 100 mL measure of a solution of ammonium carbonate ((NH_4_)_2_CO_3_, 0.1M) was added to 100 mL of a solution of calcium chloride (CaCl_2_, 0.1 M) in a glass bottle, and the pH was adjusted to 10 with concentrated ammonia, and the reaction was kept under vigorous mechanical stirring for 1 h at room temperature. The resulting mixture was centrifuged at 6000 rpm for 10 min before washing with ethanol. The collected solid product was dried in the oven for 24 h at 60 °C. The obtained materials are referred to as V.

### 4.2. Synthesis of Manganese Carbonates (One-Pot Synthesis)

A 100 mL measure of an ammonium carbonate solution ((NH_4_)_2_CO_3_, 0.1 M) was added to a combined solution of 50 mL of a calcium chloride solution (CaCl_2_, 0.1 M) and 50 mL of a manganese chloride solution (MnCl_2_, 0.1 M) in a glass bottle. The pH of the mixture was then adjusted to 10 with concentrated ammonia, and the reaction was kept under vigorous mechanical stirring for 1 h at room temperature. The reaction mixture was then centrifuged at 6000 rpm for 10 min, and the resulting pellet was then washed with ethanol. The final collected solid product was dried in the oven for 24 h at 60 °C. The obtained materials are referred to as Mn-V.

### 4.3. AP Ligand Functionalization

For the incorporation of the 3-aminopropyltriethoxysilane (AP, 2.4 g, 10.8 mmol) ligand, the reaction was carried out as follows. In short, an AP ratio of 1:2 by mass with the material was established. In a Schlenk flask, 1.2 g of vaterite was added, and the material was subsequently dispersed in 25 mL of toluene before final addition of the ligand. This was left to react at 60 °C for 72 h under stirring. Afterward, the mixture was centrifuged at 6000 rpm for 10 min, and the solid precipitate was washed with ethanol. Finally, the solid product was kept in the oven for 24 h at 60 °C. The obtained materials are referred to as V-AP.

### 4.4. Cysteine Functionalization

For functionalization with 10% cysteine, the following EDAC coupling reaction was carried out. In a glass vial, 50 mL of MES buffer (0.1 M) was added together with EDC hydrochloride (EDAC, 118.29 mg, 0.62 mmol) and N-hydroxysuccinimide (NHS, 177.48 mg, 1.54 mmol). The mixture was left for 5 min under stirring. After this time, cysteine (cys, 50 mg, 0.41 mmol) was added and left under stirring for 15 min to activate the carboxyl group of the amino acid. Finally, V-AP/Mn-V-AP material (500 mg) was added and left to react for 2 h at room temperature. Subsequently, the suspension was centrifuged at 6000 rpm for 10 min and washed with ethanol. The obtained product was kept in the oven for 24 h at 60 °C. For functionalization with 25% cysteine, the reaction was carried out using the same procedure, but adding 125 mg (0.41 mmol) of the amino acid, 295.59 mg (0.62 mmol) of EDAC, and 443.65 mg (1.54 mmol) of NHS. The obtained materials are referred to as V-AP-cys10, V-AP-cys25, Mn-V-AP-cys10, and Mn-V-AP-cys25.

### 4.5. Manganese Functionalization

For the post-synthesis method of Mn incorporation, the following procedure was used. In a glass flask, 150 mg of V-AP-cys with 54.04 mg of manganese chloride (MnCl_2_, 197.91 g/mol) was added, and then the mixture was suspended in dimethylformamide (DMF, 20 mL). The mixture was reacted with vigorous stirring for 24 h at 60 °C. Subsequently, the resulting suspension was centrifuged at 6000 rpm for 10 min, and the resulting solid was washed with ethanol. The final solid products (V-Ap-cys10-Mn and V-AP-cys25-Mn) were kept in the oven for 24 h at 60 °C.

### 4.6. Characterization Techniques

FT-IR measurements were carried out in a PerkinElmer Spectrum Two FT-IR spectrophotometer (PerkinElmer, Waltham, Massachusetts, USA) between 4000 and 400 cm^−1^, using KBr pellets. UV–Vis measurements in solids were carried out on an FT-IR Spectrum two (Perkin Elmer) spectrophotometer (Agilent Technologies, Santa Clara, CA, USA) with an integrating sphere and polytetrafluoroethylene (PTFE) as reference. For the powder X-ray diffraction analysis, the Philips PW3040/00 X’Pert MPD/MRD spectrometer (Philips, Amsterdam, The Netherlands) was used, with monochromatic radiation. X-Ray fluorescence was carried out with a Panalytical MagiX X-ray fluorescence spectrometer Model MagiX (Malvern Panalytical, Malvern, UK). Transmission electron microscopy was carried out on a JEOL JEM 1010 operating at 100 kV (JEOL Ltd., Tokyo, Japan). DLS measurements was carried out on a Nanoplus (Micrometrics) using PBS pH 7.4 as dispersion medium. Inductively coupled plasma mass spectrometry (ICP-MS) measurements of Mn were recorded using an Agilent 7700 spectrometer (λ_Mn_ = 257.610 nm).

### 4.7. Cell Assay

Murine glioblastoma (CT2A) and breast cancer (SKBR3) cells were used. These were obtained from the American Type Culture Collection (ATCC, Manassas, VA, USA). These were cultured in Dulbecco’s modified Eagle’s medium (DMEM-High Glucose, Dominique Dutscher, Bernolsheim, France) with 10% fetal bovine serum (FBS, PAN Biotech, Aidenbach, Germany), 2 mM L-glutamine (FBS, PAN Biotech, Germany), and 1% penicillin/streptomycin (Corning, New York, NY, USA) at 37 °C in a humidified incubator (Series II water Jacker, Thermo Scientific, Waltham, MA, USA) with 5% CO_2_.

To carry out the dose–response curves and establish the levels of cell cytotoxicity, 96-well plates (p-96) were used. The plates were divided in such way that 3 concentrations could be tested as well as a positive control, with 4 replicates and 3 independent assays; plates were optimized to be able to test 6 materials per experiment.

The cells were seeded at a concentration of 200,000 cells/mL in p-96 and were left in the incubator for 24 h at 37 °C with 5% CO_2_, after which time the compounds to be evaluated were added at 1, 0.5, 0.25, and 0 mg/mL (negative control) before being left to incubate for 72 h at 37 °C with 5% CO_2_.

The toxicity assay used was a Thiazolyl blue tetrazolium bromide (MTT) assay (BioChemica, Spain). After 72 h, the p-96 was emptied and a 0.5 mg/mL MTT solution was added. The plate was left in the incubator for 4 h at 37 °C with 5% CO_2_. After that time had elapsed, it was emptied, and DMSO (Labbox Labware, S.L., Barcelona, Spain) was added to solubilize tiazol precipitations. The plate was read in the spectrophotometer BIOBASE-EL10A (Biobase, Jinan, China) at 542 nm.

To verify the internalization of the materials in the cells, CT2A, SKBR3, and 293T cells were exposed to 0.25 mg/mL (concentration with respect to vaterite) of the compounds Mn-V-AP and Mn-V-AP-cys25, repeating the incubation conditions mentioned above. Cell lysis was then performed with 1X RIPA Lysis Buffer (Thermo Scientific™, Waltham, MA, USA) according to the supplier’s specifications. The intracellular content of manganese was measured using ICP to determine the amount of cell internalization (Table S1 of Supplementary Data).

### 4.8. Statistical Analysis

A one-way analysis of variance (ANOVA) was used to compare the groups and determine the differences. A *p*-value < 0.05 was employed in all tests to indicate statistical significance. GraphPad Prism 6 (GraphPad Software Inc., San Diego, CA, USA) was employed to perform the statistical analyses.

## 5. Conclusions

The present study includes an analysis of the anticancer activity of the different functional modifications of vaterite in glioblastoma multiforme, one of the most resistant cancer types, with the tested materials managing to reduce cell viability by more than 50% when using a combination of manganese with cysteine. Incorporation of cysteine seems to induce selectivity of the systems towards cancer cell lines, while loading of the materials with Mn improves the cytotoxic nature of the systems. Additional studies were carried out on breast cancer cells, obtaining comparable results. The results presented here are promising, as they provide future contributions to combat highly resistant tumors that do not respond to current treatments.

Future studies from our team will focus on testing the reported systems against primary cultures of different cell types to move further to in vivo tests in animal models. This will allow us to observe whether the Mn-modified vaterite-based materials incorporating cysteine could also be subsequently loaded with some other therapeutic agents to be used as enhanced drug-delivery systems with potential applications in clinical oncology.

## Figures and Tables

**Table 1:** Crystallite size of all vaterite materials.

Material	D (nm)
V	62.41
V-AP	49.06
V-AP-cys10	57.82
V-AP-cys25	53.24
V-AP-cys10-Mn	50.90
V-AP-cys25-Mn	59.62
Mn-V	16.49
Mn-V-AP	7.95
Mn-V-AP-cys10	9.34
Mn-V-AP-cys25	9.49

**Table 2:** Percentage of Cysteine and Manganese in the V and Mn-V series.

Material	S		Cysteine		Manganese	
	Concentration (%)	Absolute Error (%)	Concentration (%)	Absolute Error (%)	Concentration (%)	Absolute Error (%)
V-AP-cys10	0.009	0.001	0.036	0.004	n.a. ^b^	n.a. ^b^
V-AP-cys25	0.027	0.001	0.104	0.004	n.a. ^b^	n.a. ^b^
V-AP-cys10-Mn	n.a. ^a^	n.a. ^a^	n.a. ^a^	n.a. ^a^	0.41	0.02
V-AP-cys25-Mn	0.015	0.001	0.058	0.004	0.090	0.004
Mn-V	n.a. ^b^	n.a. ^b^	n.a. ^b^	n.a. ^b^	36.80	0.09
Mn-V-AP-cys10	0.177	0.003	0.67	0.01	24.60	0.08
Mn-V-AP-cys25	0.191	0.003	0.72	0.01	22.70	0.08

^a^ Not analysed. ^b^ Not applicable.

**Table 3:** Hydrodynamic particle size and polydispersity index.

Material	D (nm)	PDI
V	1183 ± 203	1.61
V-AP-cys25	1078 ± 165	0.86
Mn-V	1541 ± 515	1.13
Mn-V-AP-cys25	1559 ± 492	1.57

## Data Availability

Not applicable. All data are available in the main text.

## Supplementary Materials

The following supporting information can be downloaded at https://www.mdpi.com/article/10.3390/ijms24065546/s1.
