# Supplementary material for: New Carbonate-Based Materials and Study of Cytotoxic Capacity in Cancer Cells
Source: Int J Mol Sci. 2023 Mar 14;24(6):5546. doi: 10.3390/ijms24065546 (PMC10055759; doi:10.3390/ijms24065546)
Supplement: Supplementary file 1 [file ijms-24-05546-s001.zip › ijms-2213220-supplementary.pdf]

Supplementary data

New Carbonate-Based Materials and Study of Cytotoxic Capacity in Cancer Cells

Nayara Niza-Pérez 1,†, Josefa Quiroz-Troncoso 2,3,†, Nicolás Alegría-Aravena 2,4,5, Santiago Gómez-Ruiz 1, Diana Díaz-García 1,* and Carmen Ramírez-Castillejo 2,6,*

1 COMET-NANO Group, Departamento de Biología y Geología, Física y Química Inorgánica, ESCET, Universidad Rey Juan Carlos, 28933 Madrid, Spain
2 Centro de Tecnología Biomédica (CTB), Departamento Biotecnología-B.V. ETSIAAB, Universidad Politécnica de Madrid, 28223 Madrid, Spain
3 Medicinal Gardens S.L. (Baïa Food), 28008 Madrid, Spain
4 Grupo de Biología y Producción de Cérvidos, Instituto de Desarrollo Regional, Universidad de Castilla-La Mancha, 02006 Albacete, Spain
5 Asociación Española Contra el Cáncer (AECC)—Fundación Científica AECC, 02001 Albacete, Spain
6 Oncology Department, Instituto de Investigación Sanitaria San Carlos (IdISSC), 28040 Madrid, Spain
* Correspondence: diana.diaz@urjc.es (D.D.-G.); mariadelcarmen.ramirez@upb.es (C.R.-C.); Tel.: +34-910679290 (C.R.-C.)
† These authors contributed equally to this work.

This supplementary data contains:

1) Table S1. Concentration of intracellular Mn and percentage of internalization in the three cell lines.

Table S1. Capacity of selectivity (internalization) of some studies materials measured by ICP.

	Cell lines					
	CT2A		SKBR		HEK-293T	
Material	[Mn] intracellular (ppm)	% Real Internalization	[Mn] intracellular (ppm)	% Real Internalization	[Mn] intracellular (ppm)	% Real Internalization
Mn-V-AP	4.00	33.44	3.80	31.77	4.00	22.44
Mn-V-AP-cys25	4.20	52.86	4.10	51.60	4.10	51.60
